# Usefulness of an Endodontic Case Difficulty Assessment Form of Root Canal Treatments in Dental Education in Finland

**DOI:** 10.3390/dj9100118

**Published:** 2021-10-14

**Authors:** Reetta Pesonen, Tarja Tanner, Taina Käkilehto, Kristiina Oikarinen-Juusola, Marja-Liisa Laitala, Vuokko Anttonen

**Affiliations:** 1 Research Unit of Oral Health Sciences, University of Oulu, P.O. Box 5281, 90014 Oulu, Finland; tarja.tanner@oulu.fi (T.T.); kristiina.oikarinen-juusola@fimnet.fi (K.O.-J.); marja-liisa.laitala@oulu.fi (M.-L.L.); vuokko.anttonen@oulu.fi (V.A.); 2 Medical Research Center, Oulu University Hospital, University of Oulu, P.O. Box 5281, 90014 Oulu, Finland; 3 Dental Teaching Unit, 90220 Oulu, Finland; taina.kakilehto@ouka.fi

**Keywords:** case difficulty assessment form, dental education, endodontics, evaluation, root canal treatment

## Abstract

According to current care guidelines, it would be beneficial to evaluate the difficulty of a root canal treatment (RCT) after the decision of an indicated RCT. For this matter, several difficulty assessment forms have been developed. In this pilot study, fifth-year dental students evaluated the usefulness of the Endodontic Case Difficulty Assessment Form (ECAF) presented in the Finnish Current Care Guidelines for Endodontic Treatment (2014). Another aim was to postoperatively investigate how well the evaluation by dental students using the ECAF associated with the outcome of RCT evaluated by a specialist in endodontics. The dental students’ (*n* = 33) and the supervisor’s assessments of the RCTs were compared postoperatively at the Dental Educational Unit, Oulu, Finland. After completing the ECAF, the students’ experiences of its use were explored with a structured form. In ECAF, patient-derived factors, such as gagging, deviant crown morphology, and complications in previous endodontic treatment, were all significantly associated with complications in RCTs by the dental students (*p* < 0.05). The assessments by students and the supervisor differed in 55% of cases, especially in moderately difficult cases. In the majority of these cases (71%), the students evaluated the case to be easier than the teacher. Students found the ECAF user-friendly, even if it did not demonstrate their competence in accomplishing RCTs. The ECAF appears useful for junior dentists, specifically in terms of distinguishing the least and most difficult cases. A simpler form could be useful for students and clinicians.

## 1. Introduction

Root canal treatments (RCT) were the cause of the majority of patient dental treatment complaints made to authorities in Finland between 2002 and 2015. They accounted for 31% of all of the reported healthcare-related dental reclamations [1]. This may be because RCTs are technically challenging but also because general practitioner dentists treat endodontic cases beyond their skill level. According to the European Society of Endodontology (ESE), dentists graduating in Europe must be able to recognize the factors that possibly complicate endodontic cases [2].

An endodontic case assessment form including all of the relevant factors could improve the quality of root canal treatments because advanced evaluation of case difficulty enhances the recognition of the risks that may occur during treatment [3]. Therefore, after the decision on an indicated RCT has been made, it would be beneficial for the dentist to routinely evaluate the difficulty of the case. This might prevent iatrogenic errors and facilitate case planning and decision-making; for example, this may help to determine whether the patient needs to be treated by a specialist. This is of the most importance for dentists with only a little experience. Once the difficulty of a RCT is evaluated, informing the patient about the course of treatment and its possible complications, restrictions, and costs can be achieved more specifically and accurately. Information regarding the level of difficulty of a RCT case obtained by using an assessment form also facilitates documentation [4].

Various international endodontic organizations have developed their own forms for assessing the difficulty of RCTs. However, there are only few studies on how frequently they are used and how helpful they are [5]. The majority of studies have explored the American Association of Endodontists’ (AAE) Endodontic Case Difficulty Assessment Form [6]. For example, Alamoudi et al. [7] recently concluded that the AAE’s form can help students and general practitioner dentists evaluate the level of difficulty associated with each case and thus minimize the risk of iatrogenic errors, which they found to be significantly correlated with case difficulty. Similarly, Haug et al. [8] found that the AAE’s form is an important and valuable tool in undergraduate dental education for learning to predict potential endodontic mishaps and to estimate the number of treatment visits.

The endodontic case difficulty assessment form used in this study (ECAF) is included in the Finnish Current Care Guidelines for Endodontic Treatment [9]. The form has been modified using the American Association of Endodontists’ form as a model. The European Society of Endodontology also recommends the use of either the AAE’s form or the Dutch Endodontic Treatment Index (DETI) for endodontic treatment protocols [2]. The DETI contains 15 criteria or factors contributing to the difficulty of the RCT. It has been found that the DETI form enables dentists to differentiate simple/uncomplicated cases from complicated ones [5].

According to a report by the AAE, the ability of dental student to assess endodontic case difficulty is more efficient with an endodontic case assessment form than without it [4]. The objective of this study was to explore the usefulness of the endodontic case difficulty assessment form (ECAF) presented in the Finnish Current Care Guidelines for Endodontic Treatment among fifth-year dental students. In addition, this study explored the student’s and the teacher’s experiences of using of the ECAF.

## 2. Materials and Methods

There is a specific protocol for endodontic treatment in the Dental Educational Unit of the City of Oulu, Finland. This protocol is also followed in the patient cases of the present study. Teacher supervision is available. Indications for endodontic treatments are pulpal and periapical infections.

**Figure 1:**
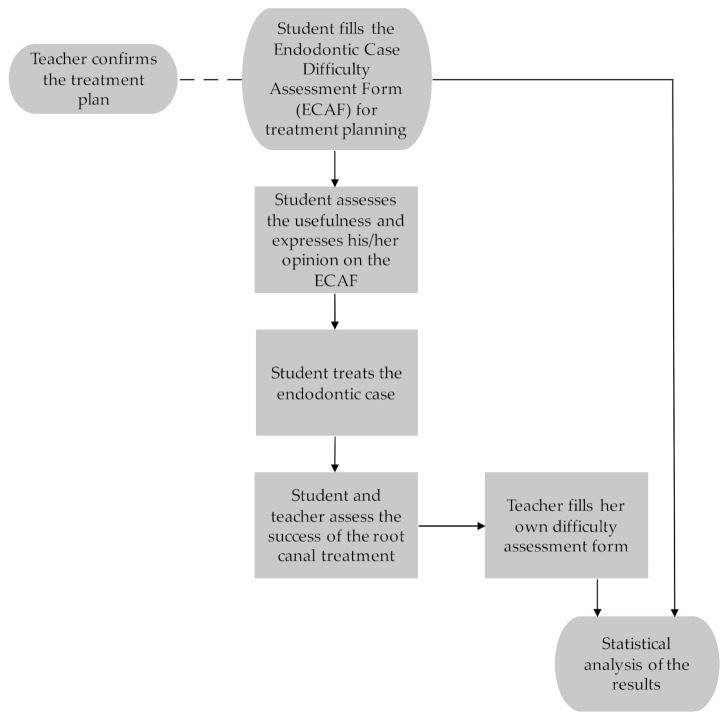
The protocol of the research arrangements.

The use of the ECAF is part of the endodontic curriculum for fifth-year dental students at the University of Oulu, Finland. The students participating in the present study were instructed to use the ECAF for treatment planning at the baseline of a RCT. The teacher responsible for endodontics at the educational clinic of City of Oulu (golden standard) who is an endodontist and an experienced clinician, confirmed the treatment plan made by the student. The same teacher filled in a teacher assessment form after the treatment, where she assessed the case difficulty according to the progression of the case and medical records. The research material consisted of case difficulty assessment forms completed by 33 students. The protocol is described in Figure 1.

The ECAF is based on a form developed by the AAE, and it was translated into Finnish when the Finnish Current Care Guidelines for Endodontic Treatment were being worked on [9]. It is a modified version of the AAE form and differs from the original one in some difficulty-affecting factors. A few factors (very long tooth >25 mm, mandibular premolar or anterior with 2 roots, combined endodontic/periodontic lesion) are classified as moderately difficult instead of being highly difficult. One factor (avulsion) is added, and one modified (wide or full coverage restoration) in the moderately difficult class, and some (general symptoms, CBCT or other special imaging needed, root apex in immediate proximity of mandibular canal, difficult endodontic retreatment) are added into the highly difficult class and are thus missing from the original version of the form. One factor (root amputation prior to endodontic treatment) that is classified as highly difficult in the AAE form is missing from the ECAF. The ECAF also differs from the AAE form in the sense that the difficulty factors are scored, and a sum score can be calculated, indicating the final level of difficulty of a case. The consensus on coefficients indicating the difficulty (1, 2 and 5) was reached by the group of experts finalizing the Finnish Current Care Guidelines for Endodontic Treatment. There are also several factors in the highly difficult category marked with an asterisk, meaning that they automatically make the case highly difficult, regardless of the sum score obtained. Factors affecting the level of difficulty of the required endodontic treatment are categorized as follows: patient related factors, diagnostic and treatment related factors, and other notable factors. Each factor belonging to each class was classified by levels of difficulty and were scored as follows: minimally difficult = score 1, moderately difficult = score 2, highly difficult = score 5. The levels of difficulty characterize the patient’s general health, nature of the endodontic work, and common risk factors. A sum, i.e., difficulty score according to ECAF, is formed on the basis of responses to the form. The difficulty categories are the following: class I = sum score 18–24 (minimally difficult), class II = sum score 25–30 (moderately difficult), class III = sum score over 30 (extremely difficult). During the analysis of the results, a new class of difficulty 0 (not difficult) was added to the study in order to also analyze cases where the difficulty scores given by the students were below the lowest score in the class I (sum score 18).

The supervisor assessed the difficulty of every RCT using a teacher’s assessment form after the treatment. This form consisted of four classes defined by different criteria, which are described in Table 1. For the analyses, the supervisor’s difficulty assessment score of 1 (simple) was decided to correspond to a student’s difficulty assessment sum scores of 0 and I. Furthermore, the supervisor’s assessment scores of 2 (challenging, but the student should be able to manage the case independently) and 3 (challenging, but the student should be able to manage the case with supervision) were decided to correspond to a student’s difficulty sum score of II. The supervisor’s assessment score of 4 (very challenging and the student should able to manage it with assistance) was decided to correspond to a student’s difficulty sum score of III.

The student assessed the technical success of the RCT together with the teacher using a structured form designed for the assessment of the success rate of the RCT. This form consisted of the duration of the RCT in months, from the diagnosis to the filling of the canals, the length of the filling (0–2 mm from the radiological apex/short/overfilled), and the quality of the filling (tight/sparse). In addition, the student had the opportunity to describe the possible complications with open comments.

The complications that appeared during treatment were classified as follows: no complication/perforation or other complications during the RCT/extraction/retreatment. Complications such as vast preparation, a canal that could not be opened, or a needle perforating the apex in the radiograph were classified as other complications. Extraction meant that the tooth was lost due to a complication or an iatrogenic error during the follow-up period. None of the root canals were refilled during the treatment. Dry dam detaching from the tooth was not considered a complication.

The students’ experiences of using of ECAF were explored with a separate form. The students were instructed to fill in this form after they had completed the ECAF but before starting the RCT. They were asked whether they considered the ECAF user-friendly and clear, whether it made assessing the difficulty of a RCT easier, whether it brought up factors that might have an effect on the treatment, and whether it clarified the student’s own potential to treat the patient. In addition, the form was designed to explore whether the students were going to use the ECAF in the future. All of these questions had three options: yes/no/I cannot answer.

### Statistics

Cross-tabulation was used to investigate the association between the difficulty assessments of the students and the supervisor as well as the outcome of the treatment, i.e., complications in association with difficulty estimations and the factors listed in the ECAF. The statistical significance of the differences between the groups was analyzed by the Chi Square test. A *p* value < 0.05 was considered statistically significant. The variables were dichotomized for the analysis of the results.

For the statistical analysis, SPSS statistical analysis software (version 25.0, SPSS, Chicago, IL, USA) was used.

## 3. Results

The students gave their assessments on 33 root canal treatments using the ECAF. Of these, the teacher’s assessment was obtained in 31 cases (94%).

One in four of the participants represented ASA class 2 (Table 2). Patient-derived factors complicating the root canal treatment such as fear, mouth opening restrictions, and gagging were discovered in more than 40% of the patients. Of these, the majority (79%) had only one complicating factor. Of all of the teeth that were treated, 70% were molars, and of these, 39% were second or third molars, and almost 40% were inclined or rotated (<30°), or tooth restoration was needed preoperatively for moisture isolation. With regard to crown morphology, there were challenges in almost 70% of the cases. One third of the cases were retreatments (*n* = 11); of these, 27% had had previous complications, and in 18%, the previous RTC had been challenging. The reported symptoms and clinical and radiologic conditions were compatible in nearly all cases (97%). Of the cases where complications occurred, 62% presented with ASA class 2, 62% had a wide restoration, and 54% were first molars (Table 2). Table 2 shows also the presence of factors affecting the RCT in the complications.

**Figure 2:**
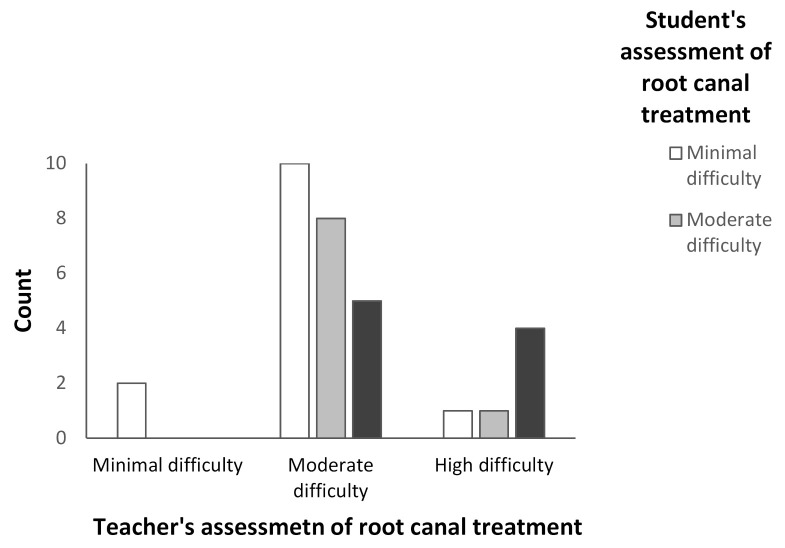
The students’ and the teacher’s assessments of the difficulty of root canal treatments.

The outcome assessment on the difficulty of a root canal treatment by the student and the supervisor differed in more than half (55%) of the cases (*p* = 0.016); generally, the students evaluated the case to be easier than the supervisor (Figure 2). On the other hand, in five cases, the supervisor evaluated the case to be moderately difficult, while the students assessed the case to be extremely difficult. The students assessed nearly half (42%) of the cases to be minimally difficult and approximately one third (29%) to be moderately difficult, whereas the teacher assessed only 7% of the cases to be minimally difficult and 74% to be moderately difficult. The greatest difference between the assessments of the students and the teacher appeared to be in the moderately difficult cases.

Forms assessing the success rate of the RCT were returned filled in concerning 88% of the cases. Complications occurred in 45% of the cases. In 69% of the cases where complications were seen, the assessments by the student and the supervisor differed. Approximately one half (46%) of the complications appeared in cases where the student had assessed the case to be easier than the teacher, whereas complications occurred in only 14% of the cases where the teacher’s and the student’s difficulty assessments were identical.

Patient-derived factors were significantly related to complications observed during the RCT (*p* = 0.010). Specifically, gagging patients had significantly more complications than other patients (60% vs. 36%; *p* = 0.033) (Table 2). Patients with abnormal crown morphology also had significantly more treatment-derived complications than those with normal tooth morphology (66% vs. 0%; *p* = 0.014). On the other hand, root curvature or anatomy in radiographs was not significantly correlated with complications. The patients who had had complications during previous RCTs had more complications (100% vs. 33%; *p* = 0.022). There was no significant correlation between the difficulty assessments and the length (*p* = 0.162) or the quality of the filling (*p* = 0.659).

Table 3 shows that the majority of the students considered the form to be easy and clear to use. The students thought that the form taught them about factors that may affect the difficulty of the RCT. The majority of the respondents reported being willing to use the form in the future. On the other hand, only a little over half of the students felt that the form made it significantly easier to assess the difficulty of the treatment, and only less than half felt that the form clarified their own potential to treat the patient.

## 4. Discussion

On the basis of the results, assessing the difficulty of a RCT is challenging for students at the end of dental school. The large number of complaints regarding dental treatment to authorities tells us that assessing the difficulty of a RCT can be difficult for qualified dentists as well [1]. The ECAF form [9] used in this study comprises patient-related, diagnostic, and treatment-related factors with a variety of effects on the difficulty level. When using this structured form, the outcomes of the difficulty assessment by students differed significantly from those by their supervisor after the completed RCT. Generally, the students estimated the cases to be easier than the experienced endodontics specialist. There were more often complications in the cases where the students’ and the teacher’s assessments differed.

The students’ and the teacher’s assessment outcomes were only identical in less than half of the cases. In the majority of the cases, the students had evaluated the case to be easier than the teacher had. This difference was the greatest when the teacher had evaluated the case to be moderately difficult. The teacher’s difficulty assessment did not include the option “easy and managed by the student with help” because the students receive guidance during every root canal treatment situation, even when they work independently.

The study found that treatment complications were more likely to occur when the student’s and the teacher’s assessment of difficulty varied. On the other hand, the occurrence of a complication during the treatment may have had an impact on the teacher’s difficulty assessment of the treatment, which could not be taken into consideration in the analyses. However, no statistically significant association was discovered in the prevalence of complications when the student’s and the teacher’s assessment of difficulty varied. This could be due to the small size of the research material. The large number of complications seen may have been due to the fact that the endodontic cases in this research material were difficult, with cases in the second and third molars being seen.

Many of the factors included in the ECAF, such as retreatment and ASA class, are unambiguous and independent of the assessor. Some of the factors, on the other hand, are determined by subjective clinical or radiological assessment. The differences in the difficulty assessments by the students and the teacher may be caused by these factors. An example of such factors is the root canal morphology assessed from a radiograph: the radiological diagnostics of root canals is completed visually from periapical radiographs, based on the person’s skills and experience [10]. An earlier study revealed that the visual assessment of the curvature of the root canal is not accurate [11]. A surprising finding in this study was that canal and root morphology, which are normally considered to complicate the treatment, did not complicate it significantly.

Numerous factors are categorized in the highly difficult class in the ECAF. It is possible that students do not have time to fully consider all factors and may consequently miss a possible hazardous factor. An experienced dentist is probably able to manage this assessment more easily within the time limit. This indicates that there is a need to develop a simplified ECAF, prioritizing the most effective factors, to promote its use among clinicians, dental students, and dentists who have recently graduated as well.

In their article, Ree et al. [5] evaluated two different endodontic case difficulty assessment forms: the Dutch Endodontic Treatment Index (DETI) and the Endodontic Treatment Classification (ETC). According to them, the DETI can be used to quickly separate easy cases from difficult ones, whereas the ETC helps to assess the difficulty of a RCT more precisely. They are recommended to be used side by side [5]. However, using two forms may be too laborious for general dental practitioners. The form explored in this study was similar to the ETC form. However, it worked in a similar way to the DETI and could not distinguish the difficulty levels as precisely as the ETC. The ECAF appeared to be the best for distinguishing the least and most difficult cases specifically. The different results here compared to previous results found in the literature may be caused by the fact that in the study of Ree et al. [5], the ETC was used by graduated dentists who were able to benefit from the form based on their own clinical experience.

On the other hand, the use of a difficulty assessment form before a RCT could be justifiable to all general dental practitioners (GDP). Laukkanen et al. [12] found that teeth treated by dental students had a success rate of 84%, whereas the success rate for teeth treated by GDPs was only 67% [13]. The success rate was especially low for molars, only 56%.

Some questions in the ECAF form should be re-evaluated in the possible revised version of the form. The alternative that the tooth has no history of trauma should be added to the factor “trauma history”, for instance. Likewise, no pain or swelling should be added to the factor assessing emergency condition. The alternatives associated with endodontic treatment history could also be edited. A short guide on how to fill in the form could be added to increase its usefulness.

The decision to refer the patient to an endodontist depends on the skills and experience of the referring dentist and the availability of specialized dental care. Assessing one’s own skills can be challenging not only for students but also for graduate dentists. In their article, Rosenberg and Godis [3] state that referral to a specialist is indicated when an endodontic case contains multiple complicating factors or a single factor that makes treatment very challenging. There may be a higher risk for complications if a dentist chooses to treat a case that is too difficult in relation to his/her skills. Uimonen and his co-workers [14] state that endodontic cases that are referred to a specialist are expensive, which may keep dentists from referring patients. On the other hand, in the end, treatment with complications is expensive as well. It has also been reported that a single visit model may improve the productivity of dental services [15].

The ECAF form teaches students the factors that have an effect on the level of difficulty of the treatment. It is easy to use and is clear for both the students and the teacher. However, the form did not clarify the student’s own potential to treat the patient, which is ultimately the goal of the difficulty assessment. This would be especially important for the pretreatment evaluation of moderately difficult endodontic cases.

This study was not able to find any factors affecting the RCT that led to a different difficulty assessment outcome between the student and the real difficulty of the case. This could have been better explored if the teacher had filled in the same form as the students. This can be considered as a limitation of this study. Additionally, the study population was limited. The endodontic teaching setting is both an advantage and a limitation; there is enough time for evaluation, but it is not a field situation. This study provided evidence on the need for this kind of evaluation form.

The ECAF should be made simpler, but it must include the most important factors affecting the treatment: patient-related factors, deviant crown morphology, and whether there have been complications during previous endodontic treatment. The form could be used to assess the difficulty of the RCT together with the teacher and the student so that the teacher’s experience would complete the assessment and thus improve it as a learning experience. There have been promising attempts to develop a digital difficulty assessment method [16]. The Dental Practicality Index (DPI) aims to investigate the overall treatability of a tooth in terms of whether to restore, endodontically treat, refer, or extract a tooth depending on the case [17]. Developing, testing, and investigating a new simple but comprehensive modification of the ECAF that may also be in a digital format could be a topic for future studies.

## 5. Conclusions

This study adds to scarce literature on the current topic. The endodontic case assessment form (ECAF) appears to be most useful in distinguishing the least and the most difficult cases specifically. Dental students generally estimate the cases as being easier than they are. The results provided here create an opportunity to develop the ECAF further for the purposes of education and clinical practice.

## Figures and Tables

**Table 1:** Criteria of the teacher’s difficulty assessments and corresponding assessments by dental students.

Criteria for Assessing Difficulty of Root Canal Treatment (RCT) and Respective Categories			
Student (Preoperative)		Teacher (Postoperative)	
0, I (*n* = 13) ECAF < 18–24 p	Minimal difficulty	Class 1 (*n* = 2)	Easy, student managed independently without difficulties
II (*n* = 9) ECAF 25–30 p	Moderate difficulty	Class 2 (*n* = 17)	Challenging, but student managed independently
		Class 3 (*n* = 6)	Challenging, student managed with supervision
III (*n* = 9) ECAF > 30 p	Extreme difficulty	Class 4 (*n* = 6)	Very challenging, student managed it with supervision

**Table 2:** Presence of the factors affecting root canal treatment in the research population and presence of those factors in the complications during the RCT.

Type	Factor Affecting the Level of Difficulty	Presence in the Study Population (%)	Presence in the Complications (%)
**Patient related**	ASA2	42.2	61.5
	Vasoconstrictor intolerance	3.0	7.7
	Patient with fear, but treatable	15	38.5
	Restricted mouth opening	24.2	23.1
	Gagging	15.2	23.1
	Moderate pain or swelling	15.2	7.7
**Diagnosis** **and** **Treatment**	Confusing and complex signs and symptoms: difficult diagnosis	3.0	0
	Moderate difficulties in taking or interpreting X-rays	15.2	7.7
	Incisor/premolar	27.3	30.8
	First molar	42.4	53.8
	Second or third molar	27.3	15.4
	Rotated or inclined (<10°)	21.2	15.4
	Rotated or inclined tooth (10°–30°)	39.4	15.4
	At least column preparation for moisture isolation	36.4	38.5
	Wide restoration	45.5	61.5
	Pillar in the bridge Moderate deviation from normal tooth/root form	3.0 3.0	7.7 0
	Distinct loss of tooth material	27.6	38.5
	Restoration has altered the original morphology or proportions of crown and root	3.0	0
	Curved canals (10°–30°)	9.1	7.7
	Apical opening 1–1.5 mm in diameter	3.0	7.7
	Very long tooth (>25 mm)	3.0	0
	Mandibular incisor/premolar with two roots	3.0	0
	Curved canal (>30°) or S-shaped canal	9.1	7.7
	Maxillary premolar with 3 roots	3.0	0
	Canal divides in the middle or apical third	3.0	7.7
	Root apex in immediate proximity with mandibular nerve canal	3.0	0
	Abnormal view of root canals in radiograph	33.3	38.5
	Resorption	9.1	15.4
**Other**	Uncomplicated crown fracture in fully developing or immature tooth	9.1	15.4
	Complicated crown fracture in fully developed tooth	3.0	7.7
	Endodontic retreatment	33.3	38.5
	Moderate periodontal disease	15.2	30.8

**Table 3:** Distribution of fifth-year dental students (*n* = 32) according to their responses on the claims about Endodontic Case Difficulty Assessment Form (ECAF) for root canal treatments (RCT) as well as their estimation on its use in future.

The EACF…	Yes	Somewhat	I Cannot Say	A Little
	%			
is user-friendly and clear	87.5	3.1	9.4	
taught factors that may affect the difficulty of RCTs	100	0	0	
made it significantly easier to assess the difficulty of RCTs	59.4	9.4	28.8	3.1
clarified my own potential to treat the case	40.6	12.5	50	
I am going to use the form also in future	78.6	14.3	7.1	

## Data Availability

The data presented in this study are available upon request from the corresponding author.
